# Red Blood Cell Distribution Width: A Novel Predictive Indicator for Cardiovascular and Cerebrovascular Diseases

**DOI:** 10.1155/2017/7089493

**Published:** 2017-09-06

**Authors:** Ning Li, Heng Zhou, Qizhu Tang

**Affiliations:** ^1^Department of Cardiology, Renmin Hospital of Wuhan University, Wuhan 430060, China; ^2^Cardiovascular Research Institute, Wuhan University, Wuhan 430060, China; ^3^Hubei Key Laboratory of Cardiology, Wuhan 430060, China

## Abstract

The red blood cell distribution width (RDW) obtained from a standard complete blood count (CBC) is a convenient and inexpensive biochemical parameter representing the variability in size of circulating erythrocytes. Over the past few decades, RDW with mean corpuscular volume (MCV) has been used to identify quite a few hematological system diseases including iron-deficiency anemia and bone marrow dysfunction. In recent years, many clinical studies have proved that the alterations of RDW levels may be associated with the incidence and prognosis in many cardiovascular and cerebrovascular diseases (CVDs). Therefore, early detection and intervention in time of these vascular diseases is critical for delaying their progression. RDW as a new predictive marker and an independent risk factor plays a significant role in assessing the severity and progression of CVDs. However, the mechanisms of the association between RDW and the prognosis of CVDs remain unclear. In this review, we will provide an overview of the representative literatures concerning hypothetical and potential epidemiological associations between RDW and CVDs and discuss the underlying mechanisms.

## 1. Introduction

Cardiovascular and cerebrovascular diseases (CVDs) are the general terms for all heart and cerebrum diseases related to vasculopathy, which mainly include heart failure (HF), atherosclerosis, myocardial infarction (MI), arrhythmia, hypertension, and stroke [1]. Although medications and devices have been improved in recent years, mortality and morbidity in patients with CVDs remain high in recent years. CVDs continue to be the most common cause of death globally, rather than cancer and communicable diseases [2, 3]. The accurate risk assessment and stratification as well as prognosis evaluation for patients with CVDs, especially high-risk populations, are critical to cardiologists and neurologists. Current diagnosis of CVDs mainly relies on clinical judgment, imaging manifestations, and some biochemical parameters, but indexes which could be routinely used in clinic are relatively finite [4]. Ideal diagnostic markers should be highly specific and sensitive, rapidly available, inexpensive, and noninvasive [5]. The clinicians have been attempting for years to seek such a biomarker to aid identification for early prevention, intervention, and therapy to prevent adverse cardiovascular and cerebrovascular events.

**Figure 1:**
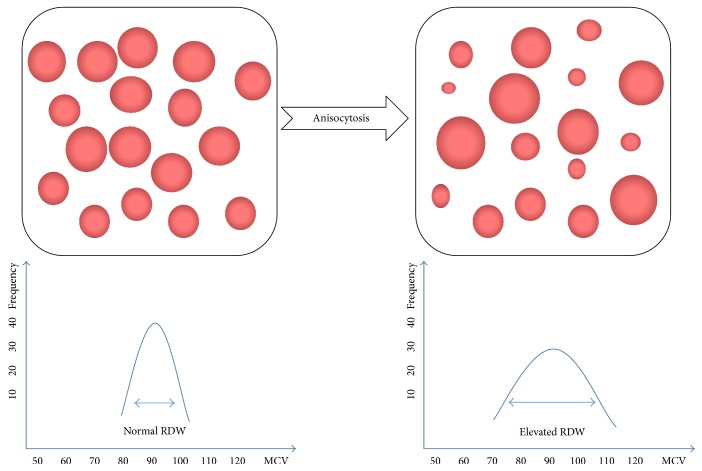
RBCs volume normal distribution curve from an impedance-based hematology analyzer and relative variation in the RBC distribution width (RDW) [40].

**Figure 2:**
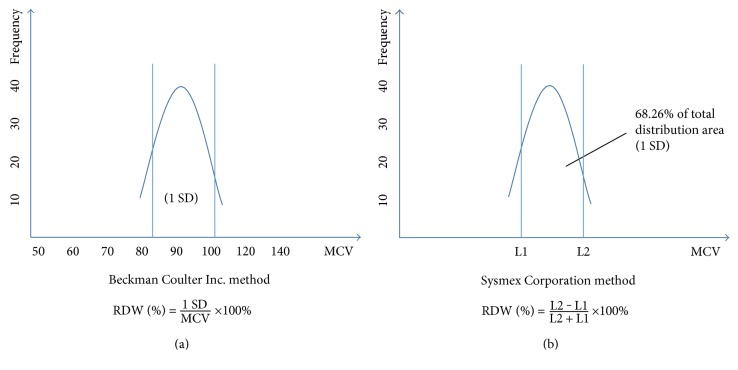
2 common methods to calculate the red blood cell distribution width (RDW) [41].

One candidate is red blood cell distribution width (RDW), obtained from a standard complete blood count (CBC). It is a measure of the variability in size of circulating erythrocytes and is indicated as the coefficient of variation of the erythrocyte size [6]. Normally, the size of red blood cells (RBCs) varies from 80 to 100 fL in the blood. Clinical conditions in which RBCs routinely elevated or decreased are usually caused by ineffective RBC production including iron deficiency, vitamin B12 and folate deficiency, increased RBC devastation, and blood transfusion [7]. The alteration of erythropoiesis can result in an extensive RBC size heterogeneity, which suggests that some pathological changes are occurring in organisms. In Table 1, we give a summary of the physiological conditions that lead to alteration of RDW values [8]. RDW is usually calculated by dividing the standard deviation (SD) of the mean corpuscular volume (MCV) by the MCV and multiplying by 100 to yield a percentage value to be on behalf of the RBC size heterogeneity [6]. Since the methods of measuring the RBC size, the instruments, the experimenters, the laboratory standards, and statistical approaches are dissimilar in different laboratories [9], there is no universal reference range till now [10]. The normal reference range of RDW most laboratories used was 11–15% [11].  Figure 1 shows RBC volume normal distribution curve and relative variation in RDW.  Figure 2 shows 2 common methods to calculate RDW.

Previous studies have presented a powerful correlation between RDW and the severity and progression in patients with CVDs, the correlation of which is even stronger than traditional risk factors. Hence, the alteration of RDW level may be a predictive indicator of morbidity and mortality for CVDs. However, the underlying mechanisms are still exceedingly unclear till now. Here, we will discuss the association between RDW and CVDs and some possible mechanisms.

## 2. RDW and Cardiovascular Diseases

### 2.1. Heart Failure

Chronic heart failure (CHF) is regarded as a systemic disease based on the chronic inflammation status with high comorbidity and mortality [12]. Accurate risk stratification of patients with CHF to screen high-risk patients is increasingly vital to target the use of evidence-based therapies efficiently [13]. The capacity of RDW in CHF to predict the onset and the short or long prognosis has been assessed thoughtfully by many studies in these years (Table 2).

In 2007, Felker et al. [14] firstly identified the potential predictive function of RDW in CHF patients. They studied 2679 symptomatic CHF patients from the North America CHARM program and assessed the relationship between routine blood tests and outcomes using a Cox proportional hazards model. Then, they found that the increased RDW was an intensely strong independent predictor of morbidity and mortality in CHF patients (adjusted hazard ratio 1.17 per 1 SD increase, *p* < 0.001). Subsequently, they analyzed another sample from the DUKE databank using the same statistic method and drew the similar conclusion finally. Notably, the RDW statistical association with CHF outcomes was higher than the widely accepted parameters of risk such as NYHA functional class, ejection fraction, and renal function but weaker than age and cardiomegaly in their study. In 2015, Ark [15] carried out a prospective study including a total of 230 consecutive acutely decompensated heart failure (ADHF) patients (149 males, 81 females). Whereafter, they divided the patients into 2 groups according to the length of hospital stay (LOS) (the demarcation is 7 days). The result indicated that high RDW levels on admission could predict prolonged LOS in ADHF patients for the first time. Interestingly, before this study, Whellan et al. [16] held the view that the risk stratification for LOS based on patient admission characteristics was limited in HF. Besides, the statistical relationship between positive RDW change from admission to 1 month after discharge and cardiovascular events in ADHF patients was also proposed. As Oh et al. [17] reported in 2012, patients with positive RDW change between admission and 1 month after discharge were more likely to have cardiovascular events compared to those without the change (60.3% versus 47.2%, log rank: *p* = 0.007). In a recent retrospective study, Liu et al. [13] evaluated the predictive value of RDW for mortality during hospitalization of CHF patients and compared it with that of NT-proBNP and other biochemical parameters. They found that RDW is indeed a predictor of mortality during hospitalization, but the predictive value is lower than that of NT-proBNP and similar to that of other biochemical parameters including lymphocyte, white blood cells, and neutrophil. This investigation suggests that the combination RDW and other validated biomarkers such as NT-proBNP can help cardiologists improve the accurate rate of diagnosis and reduce the misdiagnosis.

### 2.2. Myocardial Infarction

The World Health Organization (WHO) has classified coronary disease into five clinical types, including stenocardia, myocardial infarction (MI), ischemia HF, asymptomatic myocardial ischemia, and sudden death [18]. Coronary atherosclerosis is a chronic disease which has stable and unstable periods. People during an unstable period may develop a MI if the vascular wall is stimulated by inflammation. In a lifelong chronic disease, MI may be one minor event and even cannot be detected, but it may also be a major catastrophic event which will lead to sudden death or severe hemodynamic deterioration [19]. Apart from the routine index in clinic, higher levels of RDW have been proposed to be associated with the adverse outcomes in patients with MI (Table 3).

The first study investigating whether RDW levels were associated with risk of all-cause mortality and adverse outcomes in people with coronary disease without symptomatic HF baseline was carried out by Tonelli et al. from the University of Alberta in 2008 [20]. They performed a post hoc observational analysis of 4111 participants who were divided into pravastatin group and placebo group at random and used a cox proportional hazards model to analyze the association between RDW levels and their outcomes. Similarly, they observed a graded independent relation between high RDW levels and increased risk of all-cause death in people with coronary disease [95% confidence interval (CI), 1.05–1.24]. People with RDW in the highest quartile had a totally adjusted hazard ratio for experiencing MI of 1.43 (95% CI, 1.03–1.99). Interestingly, RDW levels of most participants in this study were high but within normal range. Meanwhile, the researchers also adjusted MCV value and found that vitamin B12 and folate were adequate in these participants. Therefore, we think nutritional deficiency seems a plausible hypothesis. Gul et al. [21] evaluated RDW levels in a total of 310 patients with non-ST elevation MI (NSTEMI) and unstable angina pectoris (UAP). Patients after discharge were followed for clinical outcomes for up to 3 years. The results showed that the 3-year mortality rate was 19% in patients with high RDW levels (RDW>14.0%, *n* = 95) versus 5.6% in patients with low RDW levels (RDW<14.0%, *n* = 215). As regards the association between RDW and all-cause mortality in patients with ST elevation MI (STEMI), Sun et al. [22] enrolled 691 patients with STEMI but free of HF. The patients with STEMI were then divided into high-RDW level group (RDW > 13.0%, *n* = 362) and low-RDW level group (RDW < 13.0%, *n* = 329). All-cause mortality rates were compared, and multivariate analysis were carried out between the two groups. They revealed that the cumulative incidence of all-cause death was significantly higher in the high-RDW group compared with the low-RDW group, and high-RDW levels were associated with all-cause mortality in patients with STEMI [hazard ratio (HR): 3.43; 95% CI 1.17–8.32; *p* = 0.025]. In a recent study, Tunçez and his coworkers [23] also reported that an RDW level over 13.9 can predict the development of stent thrombosis with a sensitivity of 57% and a specificity of 52% in patients with STEMI undergoing primary percutaneous coronary intervention.

In another large prospective study, Skjelbakken et al. [24] investigated the association between RDW and the risk of first-ever event of MI in 25,612 participants recruited from a general population. During a median follow-up time of 15.8 years, 1799 participants in total experienced a first-ever MI. Meanwhile, they found a linear association between RDW levels and the risk of first-ever event of MI, for which a 1% increment in RDW level was associated with a 13% increased risk (HR: 1.13; 95% CI 1.07–1.19). Noteworthily, participants with RDW in the lowest quintile had 71% lower risk of MI compared with those with RDW above the 95th percentile (HR: 1.71; 95% CI 1.34–2.20). This investigation firstly revealed the predictive value of RDW in the general population, enlightening physicians to pay more attention to the biochemical parameters in people without cardiovascular diseases. On the contrary, a community cohort in Taiwan reported that RDW levels were not associated with MI (HR: 1.05; 95% CI 0.65–1.68) or myocardial mortality in Taiwanese participants without cardiovascular disease in history [25]. What sparks off these differences, from our part, could be ethnicity or other residual confounders which were not distributed evenly. Nijjar Aman and his coworkers have proved that the Chinese demonstrate lower mortality rates from ischemic heart disease compared to their White and South Asia neighbors [26]. Moreover, acute MI incidence of Chinese is quite varied with age-adjusted rates, which ranges from 3.3 to 108.7 per 100,000 in males and 1.0 to 40 per 100,000 in females [27]. This may explain the inconsistent results to some extent.

Additionally, Hu et al. [28] investigated the diagnostic value of joint detection of RDW and homocysteine (HCY) variable coefficient on acute MI. They collected 300 patients with coronary heart disease, among which stenocardia took up 121 cases, HF took up 65 cases and acute MI took up 114 cases. Meanwhile, 100 healthy persons having health examination were enrolled. The result revealed that the RDW and HCY were both significantly higher in acute MI groups than in the 3 other groups (*p* < 0.05). Bujak et al. [29] found that RDW had a diagnostic value in patients admitted to the intensive care unit for chest pain suggestive of acute coronary syndrome. Combined measurements of both troponin T and RDW have allowed for diagnosing MI with greater sensitivity than the analysis of troponin T alone. This indicates that the RDW and other indices such as HCY and troponin T joint detection of acute MI has a higher sensitivity and specificity, which may greatly improve the diagnosis accuracy of MI.

### 2.3. Coronary Atherosclerosis

Atherosclerosis usually occurs in the intima of medium-sized arteries at regions of disturbed blood flow, which is triggered by an interaction between endothelial dysfunction and subendothelial retention of apolipoprotein B- (apoB-) containing lipoproteins (LPS) in focal areas of arteries [30]. As one of the most typical types in atherosclerosis, coronary atherosclerosis included a complex interaction between circulation and arterial wall components, leading a series of pathological processes such as inflammation and oxidative stress [31]. Since atherogenesis is a very slowly developing condition without any symptoms, it seems inclined to go under the radar compared to its endpoints such as MI and stroke. MI and stroke are easier to measure and quantify than atherogenesis because almost all people undergoing the two diseases will go in the hospital or die. On this account, data of the association between RDW and coronary atherosclerosis are extremely limited compared with MI and stroke in clinic (Table 4).

There are now consolidated evidences that the RDW level is higher in patients with coronary atherosclerosis compared with patients without this disease, which is also an independent and pregnant index for predicting. The first study investigating the association between RDW level and coronary atherosclerosis was carried out by Çetin in 2012 [32]. In brief, they enrolled 296 stable eligible patients undergoing coronary angiography with a suspicion of coronary artery disease, of which 209 had coronary artery disease and 87 had normal coronary artery walls without atherosclerotic lesion. They graded the stenosis in the epicardial coronary arteries into 4 subgroups considering both the extent and the severity of the lesions at the coronary angiography (<50% luminal obstruction, 1, 2, and 3 diseased vessels ≥ 50%). The study showed that RDW levels were significantly different in normal group and the subgroups determined for the extent and the severity of the coronary artery disease (normal group, 14.7 ± 1.2, <50% luminal obstruction, 15.2 ± 1.2, 1, 2, and 3 diseased vessels ≥ 50%, 15.4 ± 1.2, 15.5 ± 1.3, and 15.7 ± 1.2, *p* < 0.001, resp.). Although the author found that RDW was independent of other parameters such C-reaction protein and circulating inflammatory cells, other parameters were absent regarding B-type natriuretic peptide, plasma levels of angiotensin II, EPO, makers of oxidative stress, and some other proinflammatory cytokines. Therefore, the role of RDW in coronary atherosclerosis needs to be further explored.

### 2.4. Atrial Fibrillation

Atrial fibrillation (AF) is a common complication following MI and new-onset AF has been proposed to be associated with some adverse outcomes and a large excess risk of death in patients with MI and aortic stenosis. Therefore, early intervention for new-onset AF is a potential and promoting therapeutic strategy in AF and MI patients [33]. The present studies indicate that elevated RDW, either at baseline or at discharge, as well as the change during the treatment are all associated with adverse outcomes in patients with AF (Table 5).

Like in patients with HF and MI, the alterations of erythrocyte size are commonplace in patients with AF [34]. One of the first studies investigating the major determinations of elevated RDW levels in general population was published by Eryd et al. in 2013. The authors measured RDW levels in 27,124 participants from the general population (age 45–73 years old, 62% female), who had no history of HF, AF, MI, and stroke. During a mean follow-up of 13.6 years, a total of 1894 participants (53% male) were diagnosed with AF. By adjusting potential confounding factors such as cardiovascular diseases risk factors, nutrition intake, and some hematological parameters, they observed that the hazard ratio for incidence of AF in the highest quartile RDW level group was 1.33 (95% CI, 1.16–1.53, *p* < 0.001) compared with the lowest group. Notably, the increased risk of AF was basically unchanged after they added the dietary intake of iron, folate, and B12 to the model, and the hazard ratio for incidence of AF in the highest quartile RDW level group was 1.32 (95% CI, 1.15–1.52, *p* < 0.001) versus the lowest group [35]. In a recent study, Wan et al. prospectively observed 300 patients consecutively with AF (mean age 62.6 ± 12.9 years, 50.3% males) and collected data on all-cause mortality and incidence of major adverse events (MAEs) such as sudden death, MI, HF, renal failure, and hepatic failure. They divided the patients into four quartiles according to RDW level (Q1, ≤12.8%, Q2, 12.9–13.4%, Q3, 13.4–14.3%, Q4, ≥14.3). The results showed that there were obvious increases in both all-cause mortality (2.76, 3.98, 8.40, and 13.77 per 100 person-years in each RDW quartile and incidence of MAEs 6.46, 8.18, 13.79, and 20.27 per 100 person-years in each RDW quartile) [36].

Another study enrolling 132 patients (mean age, 60.55 ± 9.5 years, 99 male) who underwent nonemergency coronary artery bypass grafting (CABG) was carried out by Ertaş. They investigated the association between the RDW and postoperative atrial fibrillation (POAF) in patients who underwent CABG. The result revealed that the preoperative RDW levels were significantly higher in patients developing AF than in those in patients without AF (13.9 ± 1.4 versus 13.3 ± 1.2, *p* = 0.03, a sensitivity of 61% and specificity of 60%). However, they found no correlation between RDW and POAF [37].

Interestingly, in a pilot observational study including 109 patients (mean age, 66.9 ± 9.5 years, 79 male), Korantzopoulos et al. evaluated the potential correlation between RDW and POAF after cardiac surgery and drew an opposite conclusion. After cardiac surgery, 44 patients developed POAF while 55 patients still remained in sinus rhythm. The multivariate logistic regression analysis showed that RDW was the only independent predictor of POAF after cardiac surgery (OR, 1.46, 95% CI, 1.078–1.994, *p* = 0.015), while it exhibited a fairly well sensitivity (80%) and specificity (60%) [38]. Why does this conclusion differ from the one come up by Ertaş? From our perspective, the study by Ertaş was a retrospective analysis while the study by Korantzopoulos was a prospective analysis. Secondly, the study by Korantzopoulos examined more clinical, echocardiographic, and laboratory indexes and some novel indexes which were associated with AF. Finally, the age of samples was different and most importantly the study by Korantzopoulos consisted both of coronary bypass patients and patients who underwent valve or combined cardiac surgery. The three tips may contribute to the inconsistence between the two studies.

In a retrospective study, Güngör et al. evaluated RDW levels and neutrophil/lymphocyte ratio (NLR) in 117 AF patients from medical records, including 103 paroxysmal and 14 chronic AF. Compared with the control group (median 12.6%, interquartile range 12.0–13.1%), the AF group has significantly higher RDW levels (median 13.4%, interquartile range 12.9–14.1%, *p* = 0.01). Whereas, the AF group had no statistical difference in NLR compared with the control group (2.04 ± 0.94 versus 1.93 ± 0.64, resp., *p* = 0.32). This indicates that it is the elevated RDW levels, not NLR, that may be an independent risk marker for nonvalvular AF [39].

Gurses evaluated the relationship of preablation RDW levels to late AF recurrence following cryoablation. They enrolled a total of 299 patients who had symptomatic paroxysmal or persistent AF despite ≥ 1 antiarrhythmic drug(s), and the patients scheduled for cryoballoon-based AF ablation were followed up at a median time of 24 months [6–29, 40–54]. The results manifested that these patients with late AF recurrence had higher RDW levels than the patients without (14.30 ± 0.93 versus 13.52 ± 0.93%, *p* < 0.001), which suggested that high RDW levels may be one good predictor of late recurrence following cryoballoon-based AF ablation [55].

Additionally, the association between RDW and AF in the patients with hypertension was also demonstrated. Higher RDW levels were found in hypertensive patients with AF than in those without AF. Higher RDW levels in the hypertensive patients may remind the physicians of the development or presence of AF [56]. Potential association of RDW with AF history in patients with sick sinus syndrome (SSS) has been also evaluated by Korantzopoulos. Patients with symptomatic SSS undergoing dual-chamber pacemaker implantation were divided into tachy-brady (AF history) group, and other forms of SSS group and their RDW levels were compared. In the study, it turned out that RDW level was independently associated with AF history in patients with SSS and is the only parameter to have an independent association with AF [57].

### 2.5. Primary Hypertension

Primary, idiopathic, or essential hypertension is defined as high blood pressure, in which secondary causes such as renal failure, renovascular disease, pheochromocytoma, aldosteronism, or other causes of secondary hypertension or Mendelian forms (monogenic) are not present. Although we have already had a comprehensive understanding of the pathophysiology and the availability of effective treatment strategies in primary hypertension, it remains a major established and modifiable risk factor for cardiovascular disease [58]. Traditionally, hypertension is diagnosed mainly by properly measured office BP readings after the physician's clinical assessment. It has been recently reported that the RDW level is higher in patients with hypertension, especially in these nondipper hypertensive patients (Table 6).

Tanindi et al. explored the role of RDW in a healthy population (*n* = 36), prehypertensive patients (*n* = 74), and hypertensive patients who are otherwise healthy (*n* = 128), retrospectively.

After adjusting some confounding factors, they found mean RDW values were 15.26 ± 0.82, 16.54 ± 0.91, and 13.87 ± 0.94 in prehypertensive, hypertensive, and control groups, respectively (*p* < 0.05). Of note, RDW is strongly linked with systolic and diastolic blood pressures (*r* = 0.848 and *r* = 0.748, resp., *p* < 0.01), which was independent of age, inflammatory status, and anemia [59].

Sevinc et al. included 126 patients (mean age 52 years, 72 male) and evaluated the relationship of their RDW levels with NT-proBNP, 6 minute walk test, pulmonary arterial pressure, and arterial blood gases analysis in patients with pulmonary arterial hypertension. The results demonstrated that pretreatment RDW has a strong significant correlation with functional capacity, right ventricle area change, NT-proBNP, and arterial oxygen saturation and CRP (*p* < 0.05). They also observed a higher mortality in pulmonary arterial hypertensive patients who had higher RDW levels [60].

Additionally, the association between RDW and nondipping BP in hypertensive and normotensive patients was also investigated. Buyukkaya et al. graded 170 patients into four groups [normotensive-dipper (NT-D), normotensive-nondipper (NT-ND), hypertensive-dipper (HT-D), and hypertensive-nondipper (HT-ND)] and measured their RDW as well as hs-CRP levels, retrospectively. The study revealed that patients with hypertension had higher RDW and hs-CRP levels than normotensive patients. What is more, the RDW levels were significantly higher in NT-ND and HT-ND patients [61].

### 2.6. Carotid Atherosclerosis and Stroke

Carotid atherosclerosis is a complex process which is characterized by the accumulation of lipid-laden macrophages and proliferating smooth muscle cells inside the vessel wall. The advanced carotid stenosis can impair cerebral blood flow and form the emboli nest, which easily blocks cerebrovascular and triggers the ischemia injury. Several lines of evidence seemingly attest that earlier stages of carotid atherosclerosis are associated with the risk of stroke [62, 63, 64]. In clinic, the neurological impairment associated with stroke is routinely assessed by NIH stroke scale (NIHSS) and physicians usually predict the outcomes of stroke using Glasgow Outcome Scale, Barthel index, and Rankin scale [65]. Current studies have validated that RDW has a statistical association with the NIHSS scores and grading in patients with stroke (Table 7) [66].

One of these studies looking into the association between RDW levels and risk of carotid atherosclerosis was published in 2010, including 156 hypertension inpatients 60 to 85 years of age. The researchers firstly detected the carotid plaque and carotid intimal-medial thickness (IMT) as well as inner diameter (ID) using ultrasound. The patients were then divided into four categories according to RDW levels (lowest, 11-12%, low, 12-13%, high, 13-14%, and highest, over 14%). A significantly higher rate of carotid plaque with increasing RDW levels and a high baseline RDW level increased with IMT/ID ratio (95% CI, 4.54–28.59, *p* = 0.008) [67] were found in these patients. However, the target participants in this study were a small population with hypertension which is a well-known risk factor for atherosclerosis. Therefore, it could not provide abundance of evidence that the observed association between RDW levels and carotid atherosclerosis was independent of anemia.

In another study, Söderholm et al. investigated the association between sex-specific qualities of RDW and increased incidence of stroke as well as its subtypes in general population. They measured the RDW levels in 26,879 persons (male, 10,318, female, 16,561) without stroke or coronary disease history from the population-based Malmö Diet and Cancer Study. Likewise, they assessed the presence of carotid plaque and IMT using ultrasound. They developed the claim that the incidence of stroke and cerebral infraction increases with the level of RDW in individuals. The hazard ratios in the highest quartile RDW level group were 1.31 for stroke and 1.32 for cerebral infraction in comparison with the lowest quartile RDW group. In addition, they also found a slice of evidence for an association between high RDW levels and increase IMT in the common carotid artery, which has been also recognized as a risk factor in ischemia stroke [68, 69]. But this research only studied the prevalence of carotid plaque, and did not follow up the participants over time. Therefore, it said nothing about RDW and progression of carotid plaque over time in the future.

Not only that, Demir et al. proposed that RDW is a promising, easy, rapid, and inexpensive index to distinguish stroke from stroke mimics (such as multiple sclerosis and epilepsy) in young patients, with a high sensitivity (73.7%) and specificity (87.9%) [70].

A cross-sectional study including 432 consecutive patients undergoing primary ischemic stroke (within 72 h) suggested that RDW levels were significantly associated with IMT (*r* = 0.436) and were also the independent predictor of carotid artery atherosclerosis. Notably, the lowest quartile of RDW level was 3.10 (95% CI: 2.46–7.65) by comparison to the highest quartile in patients with primary ischemic stroke [71]. Furthermore, in a retrospective observational study including 316 patients undergoing thrombolysis therapy, Turcato and his coworkers also reported that patients with RDW values ≥ 14.5% had a higher risk of 1-year mortality and worse survival [72].

## 3. Mechanisms

While many studies have shown the relationships between RDW and CVDs, the pathophysiological mechanisms remain unclear. Does elevated RDW level have a direct impact on CVDs, or is it merely a marker, reflecting something else going on in the body? The existing hypothesis mainly includes the microvascular disorder, anemia, inflammation cytokines, and so on.

### 3.1. Microvascular Disorder

RBCs have many vital physiological functions in our body, including carrying oxygen and carbon dioxide as well as gas exchange between blood and tissues, which are owing to their abilities to deform and flow in the microvascular network [73]. However, alterations of osmolality under some pathophysiological conditions can decrease the ability of RBCs to deform and cause an increased RDW level, which will result in low microvascular perfusion and accentuate CVDs eventually [74]. Study shows that the deformability of RBCs in microvascular disorder decreases when the RDW level is more than 14% [75]. A clinical study from Güneş et al. has suggested that being close to the upper limit of normal osmolality range (292 to 293 mOsm/kg) could be an optimal plasma osmolality level in terms of cardiovascular prognosis in patients with HF [76]. From our perspective, the predictive role of osmolality to cardiovascular prognosis may be mediated by the deformation of RBCs, which is reflected on the RDW level.

### 3.2. Anemia

To our knowledge, patients with CHF are usually accompanied by a certain degree of anemia [77]. Furthermore, renal dysfunction secondary to HF can also lead to nutritional deficiency, which will induce insufficient erythropoiesis and anemia eventually [78]. Chronic anemia is related with left ventricular hypertrophy and HF. Patients who have chronic anemia and hemoglobin less than 10 g/dL usually present specific hemodynamic compensatory responses such as low systemic vascular resistance, high cardiac output, water and sodium retention, and decrease of renal blood flow and glomerular filtration rate. These responses may cause increased cardiac workload and consequent left ventricular remodeling [79]. Severe anemia can cause obvious heterogeneity of the RBCs size and an increased RDW level. Anemia has been recognized as a well-documented risk factor in CHF, the potential mechanisms of which include inflammatory stress, inadequate production of erythropoietin, and the impact of comorbidities [77].

In 1990s, the view that iron could protect against MI came up [80]. Although some studies have approved the hypothesis, the specific relationship between anemia caused by iron deficiency and MI is still unclear [81]. Nevertheless, if the anemia persists for a long time, cardiac output will increase to maintain adequate oxygen supply due to increase in blood volume, preload, heart rate, and stroke volume along with the decrease in afterload [82]. The coronary blood flow, the oxygen-carrying capacity, and the density of the perfused capillaries affect the distance in tissue which determines myocardial tissue oxygenation. In an animal model, Aulakh et al. [83] have demonstrated that anemia could increase the cardiac infarct size and decrease cardiac function and survival in acute MI which was induced by coronary artery ligation in rats. The value of RDW in the diagnosis of iron deficiency microcytic hypochromic anemia has been also proposed, which indirectly explains the role of RDW in MI.

Anemia has been also regarded as an independent risk factor for new onset of AF and death as well as hospitalizations among elderly patients with AF. Decreased hemoglobin was independently associated with increased cardiac events in patients with AF. And how the anemia influences RDW levels has been discussed above [84, 85].

### 3.3. Inflammatory Cytokines

Previous studies [86] have proved that inflammatory cytokines can inhibit the maturation of erythropoietin-induced erythrocyte via inhibiting the bone marrow, which is reflected by an increase of RDW partly. In MI, inflammation and oxidative stress can increase RDW level by impairing iron metabolism and narrowing down the RBCs life span to modulate the response to erythropoietin through the bone marrow.

Additionally, chronic inflammation is also proved to be the root of atherosclerosis and corresponding complications [87]. It is well known that varied causative cytokines such as TNF-*α*, IL-1b, and IL-6 are upregulated in the process of atherogenesis [88]. In atherogenesis, the upregulated inflammatory cytokines in the blood can suppress the synthesis of erythropoietin (EPO) and hemoglobin, which will cause chronic inflammation anemia [89]. Inflammatory cytokines usually regulate erythropoiesis via two pathways. One pathway is that inflammatory cytokines inhibit the transcription of EPO gene in kidney and liver. The other pathway is that inflammatory cytokines inhibit the erythroid cell mutation in the bone marrow. In one hypoxic-perfused rat kidney model, the production of EPO in kidney was suppressed by TNF-*α*, IL-1b, and IL-6 [90]. The erythropoiesis in bone marrow was lowered by inflammatory cytokines, the process of which was mainly resulted from the blockage of erythroid progenitor cell proliferation and proerythroblast maturation. The regulation of inflammatory cytokines on bone marrow erythroid progenitors makes the cells desensitized to EPO [91], which blocks the antiapoptotic and promaturation function of inflammatory cytokines [92]. Accordingly, reducing the content of EPO can inhibit the production or release of mature or immature RBCs into the blood. On the other hand, the high levels of inflammation cytokines may influence iron metabolism and bone marrow in organism [93, 94].

The mechanism behind the relationship between RDW and AF is still unclear but could be explained by a direct effect of alterations in erythrocyte volume and function on the heart. The alterations in erythrocyte volume may reflect some special pathophysiological processes that act on both erythrocytes and the heart independently. In the study from Eryd et al. [35], they found that RDW was strongly associated with a high leucocyte count. However, adjusting leucocyte count did not change the association between RDW and AF from the root, which indicated that the inflammation might work potentially in the mechanistic pathway between RDW and AF. In fact, many other studies have also proposed that AF correlated with systemic inflammation, although the association was weaker than other CVDs including HF and MI. Several inflammatory markers such as IL-2, IL-6, IL-8, TNF-*α*, CRP, and MCP-1 and mediators have been proved to be associated with the presence or the outcome of AF [84, 95, 96].

Hypertension is a widely accepted risk factor for some inflammatory diseases including atherosclerotic cardiovascular and left ventricular dysfunction. A number of soluble inflammatory markers such as TNF-*α*, IL-6, IL-1, and IL-2 are involved in the pathophysiological process of hypertension [97]. Consequently, we hypothesize that the role of RDW in hypertension may be connected with the increased inflammatory status.

Pulmonary hypertension has emerged as a major complication of serious anemia. Clinical and experimental evidence suggests that hemolysis is related to pulmonary hypertension mechanistically. Hemolysis releases plasma-free hemoglobin inactivating the intrinsic vasodilator NO and arginase-1, which depletes L-arginine, the substrate for NO synthesis. As a consequence, the deformability of RBCs significantly decreased because of the inhibition of RBC-NOS, causing a high RDW level eventually [75, 98, 99].

### 3.4. Oxidative Stress

It is reported that smoking can induce oxidative stress and elevated RDW is correlated with the number of cigarettes smoked per day as well as duration of smoking [100, 101]. Smoking has been also proved to be a well-known risk factor in MI [102]. A study has identified the stronger association between RDW and MI among smokers, which exactly supports the hypothesis that RDW level can reflect the inflammation status [24].

On the other hand, there is evidence that the role of reactive oxygen species (ROS) and oxidant stress was the genesis of hypertension. Hypertensive stimuli such as angiotensin II could promote the production of ROS in the kidney, brain, and the vasculature, contributing to hypertension. Meanwhile, increased oxidant stress in the RBCs may result in deterioration in the mechanical properties of these cells and give rise to impairments in tissue perfusion, which reflected on the increased RDW levels [103, 104]

### 3.5. Free Cholesterol

Alternatively, a more direct effect of free cholesterol may be exerted by RDW in MI patients. The pathological changes in the erythrocyte membrane that has abundant free cholesterol can lead to the accumulation of erythrocyte within the atheromatous plaque [105]. The deposition of free cholesterol from erythrocyte membrane to atherosclerotic plaques will promote atherosclerosis and thereby provide lipid rich membranes to foam cells, which is propagated by inflammatory cascade [106]. On the other hand, higher cholesterol erythrocyte membrane value is also one of the causes of the deterioration of cell deformability, which can directly affect the lifespans of circulating RBCs, and this leads to greater cellular turnover and elevated RDW levels [107]. Moreover, alterations of lipid can even decrease membrane fluidity of RBCs and lead to microcirculation disturbance. This is why patients with MI sometimes have poor tissue reperfusion following percutaneous coronary intervention (PCI).

The hypothesis is also validated by the lower level of total cholesterol from the erythrocyte membrane in patients with chronic stable angina compared with patients with acute coronary syndrome [108]. The level of cholesterol from the erythrocyte membrane has been affirmed to have a positive association with RDW level in patients with coronary disease [109]. Zhong et al. [110] also verified that the level of total cholesterol correlated with the severity of coronary artery disease. Presumably, the effect of RDW on MI might be supported by the cholesterol pathway.

### 3.6. Thrombosis

In addition, RDW and stroke were found to tend to have a strong association in population with low MCV, which suggested that an underlying mechanism could be that erythrocyte turnover became lower and hence RBC lifespan changed [111]. If the proportion of senile RBCs is larger, the MCV will decrease and RDW will increase because RBCs become smaller over time in their lifespan. As biological functions of the erythrocyte decreases over time, it will be easier for erythrocyte to adhesive to endothelial cells and form thrombosis [112, 113]. A study also validated the correlation between prothrombotic effects and high RDW, which confirmed the role of RDW in cerebrovascular diseases [114]. Prothrombotic state could be promoted by the activation of renin-angiotensin system via angiotensin II type 1a receptor, which was related with the development of atherosclerotic thrombosis. The process also elevates plasma erythropoietin level, causing the rise of RDW level. This probably explains why the RDW levels were higher in patients undergoing stroke [115, 116].

### 3.7. Nutritional Deficiency

Previous studies have showed that RDW correlated with nutrition status, such as vitamin D3, transferrin, prealbumin, and albumin negatively in HF patients. Nutritional deficiency is another potential factor affecting the RDW level in CVDs. For instance, vitamin D3 is mainly responsible for cell proliferation and erythropoiesis in bone marrow. Almost all the vitamin D3 exists in the bone marrow, the concentration of which in marrow is more than two hundred times greater than that in blood. Even a minor decrease in serum vitamin D3 levels may lead to the derangement of bone marrow erythropoiesis. Meanwhile, low vitamin D levels have been linked to inflammation, higher coronary artery calcium scores, impaired endothelial function, and increased vascular stiffness. Either a high proportion of large cells or small dense cells can induce a high RDW level. The association between RDW and incidence of AF mainly existed in patients with a low MCV but RDW was not associated with incidence of AF with the top quartile MCV level [35]. Here, we surmise that the factors associated with high MCV including alcohol intake and folate or B12 deficiency are not likely to be the cause of the present association.

### 3.8. Others

The study from Yoon et al. [117] demonstrated that a progressive rise in RDW can independently predict the mortality and cardiovascular events in patients with end-stage renal disease, and the correlation between low glomerular filtration rate, microalbuminuria, and elevated RDW levels has been also proved in previous studies [118, 119]. We speculate that renal dysfunction may be linked with many other risk factors that can elevate the RDW levels, such as nutrition deficiency, secondary anemia, inflammation, and oxidative stress. Renal dysfunction may be just a comprehensive manifestation of various pathophysiological processes. Another potential mechanism is that the neurohumoral and adrenergic systems can be activated by the reduced erythropoietin, which can stimulate the erythrocyte progenitor cells, increasing the RDW level eventually [120].

## 4. Conclusion

There is reliable evidence which is supported by many epidemiological studies that patients with CVDs are more likely to have anisocytosis and high RDW levels. While many clinical studies show relationships between RDW and various disease incidences, severity, and outcomes, in these studies analyzing the associations between RDW and CVDs, there exist some inevitable limitations, for instance, some of the studies had relatively small sample size and did not illuminate the precise mechanisms. And some of the cross-sectional or post hoc analysis had no randomized groups. Possibility of residual confounding were even not ruled out routinely in some studies. Therefore, these findings warrant validation in further studies with a more accurate experimental design.

**Figure 3:**
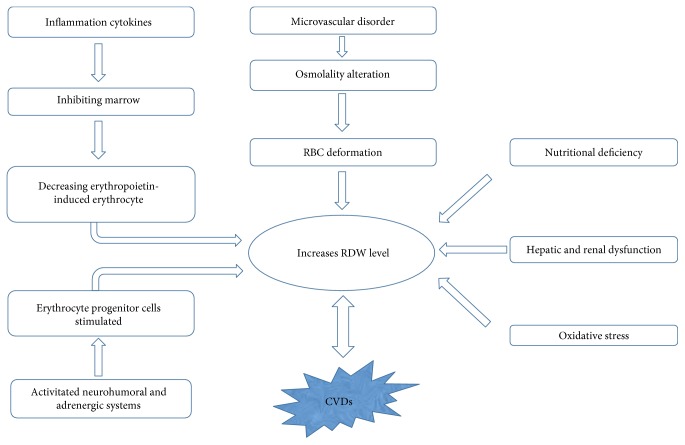
Pathophysiological process of RDW in CVDs.

We know that RDW is a numeric concept, the value of which is calculated from something else (e.g., MCV), but not something to be detected directly. Thus, we can suppose that the role of RDW to disease may be mediated by some other factors and RDW is just the combined effect of these factors. It still needs to be clarified, however, whether RDW is a simple biomarker but not a mediator in CVDs. In fact, identification of the putative causative mechanism is impeded by the lack of support from epidemiologic studies which can demonstrate whatever association exists between CVDs and anisocytosis. Here, we give a figure to show the rough mechanisms of pathophysiological process between RDW and CVDs (Figure 3). Apart from the CVDs discussed above, RDW has a distinct impact on various pathophysiological mechanisms in diseases ranging from MI to dementia. The predictive roles of RDW in other diseases including acute pancreatitis, diabetic nephropathy, Hodgkin lymphoma, and upper gastrointestinal hemorrhage have also been proposed [121, 122, 123].

In clinic, RDW is easily available to physicians as a parameter of the CBC and therefore does not incur additional costs in comparison to other clinical biomarkers in CVDs. Although the specificity of RDW is not very high, the joint detection of RDW with other index, such as NT-proBNP, cTnI, and CRP can help physicians improve the efficiency of diagnosis in CVDs because of its high sensitivity. Hence, the detection of RDW can be launched in virtually all clinical labs routinely.

Not only RDW, more and more novel biomarkers including platelet distribution width, mean platelet volume, C-reactive protein, neutrophil-to-lymphocyte ratio, and uric acid are also simple methods to assess the status of CVDs [124]. These biomarkers might be useful and promoting in clinical practice in the future. Furthermore, it will be helpful if the physicians could evaluate how much time they spend in measuring RDW, since the delays from blood sampling can make the RDW measurements abnormal. In the meantime, if different centers can develop a uniform standard for the clinical practice, the full value of RBC will be given.

## Figures and Tables

**Table 1:** Physiological conditions that lead to alteration of RDW values.

RDW values	Normal	Elevated
Decreased MCV	(i) Anemia of chronic disease (ii) Hemoglobin E trait (iii) Heterozygous thalassemia	(i) Iron deficiency (ii) HbS/Beta thalassemia (iii) Hemolytic anemia (iv) Microangiopathic hemolytic anemia
Elevated MCV	(i) Chronic liver disease (ii) Aplastic anemia (iii) Chemotherapy/antivirals/alcohol	(i) Vitamin B12, folate deficiency (ii) Immune hemolytic anemia (iii) Hereditary spherocytosis
Normal MCV	(i) Anemia of chronic disease (ii) Acute blood loss or hemolysis	(i) Early vitamin B12, folate deficiency (ii) Sickle cell anemia (iii) Transfusions (iv) Chronic hepatobiliary disease

**Table 2:** Studies exploring association between red blood cell distribution width (RDW) and heart failure (HF).

First author, journal, year	Study design	Study population	Mean follow-up	Major outcomes	Major limitations
(i) Felker et al. [14] (ii) Journal of the American College of Cardiology (iii) 2007	Retrospective cohort	2679 symptomatic chronic heart failure patients, mean age ≥ 60 years	A median of 34 months	HR for morbidity and mortality (1 SD increment of RDW): 1.17 (95% CI, 1.03–1.20)	(i) Not provide a formal evaluation (ii) Not focus on model performance
(i) Allen et al. [42] (ii) Journal Cardiac Failure (iii) 2010	Prospective, multicenter cohort	1016 heart failure patients, age 64 ± 14 years	At least 2 years	HR for hospitalization or mortality (1 SD increment of RDW): 1.06 (95% CI, 1.02–1.10)	(i) The mean follow-up was short at 1 year (ii) The research cannot distinguish causality from association
(i) Borné et al. [43] (ii) European Journal Heart Failure (iii) 2011	Population-based cohort study	26,784 subjects, age 45–73 years, without history of heart failure, stroke or myocardial infarction	A mean of 15 years	HR for heart failure in the top quartile of RDW (1 SD increment of RDW): 1.47 (95% CI: 1.14–1.89)	(i) Lack of information on type and cause of heart failure (ii) Biomarker data were only available for a subgroup
(i) Celik et al. [44] (ii) The Kaohsiung Journal of Medical Sciences (iii) 2012	Cross-sectional study	71 diastolic heart failure, age 57.09 ± 7.43 years and 50 control, age 56 ± 7 years	—	RDW > 13.6% and NT-proBNP > 125 pg/mL have high diagnostic accuracy in diastolic heart failure patients	(i) The sample size was relatively small (ii) Not measure left ventricular pressures directly
(i) Dai et al. [45] (ii) Experimental and Therapeutic Medicine (iii) 2014	Prospective cohort	521 patients with acute congestive heart failure, mean age ≥ 60 years	A mean of 24 months	Higher RDW values in acute congestive heart failure patients at admission were more prognostically relevant than Hgb levels	(i) The sample size was relatively small (ii) Not ensure that RDW is independent of Hgb levels
(i) Mawlana et al. [46] (ii) ISRN Pediatrics (iii) 2014	Cross-sectional study	31 children with heart failure, mean age 16.16 ± 14.97 months	—	RDW level was significantly related to hemoglobin level (*p* = 0.047), fraction shortening (FS) (*p* = 0.003), A (*p* = 0.12), and E/A ratio (*p* = 0.012) in children patients	(i) The sample size was relatively small (ii) No follow-up of the patient as regards (iii) Frequent hospitalization and or death
(i) Al-Najjar et al. [47] (ii) European Journal of Heart Failure (iii) 2009	Prospective cohort	1087 patients referred to a community HF clinic, age 64–78 years	52 months	Both RDW and NT-proBNP were independent prognostic (RDW: chi square 21.8 versus 49.1 both *p* < 0.001)	(i) Predictive power was examined only at a single time point (ii) No information on mode of death nor on hospitalization (iii) Not ensure that RDW is independent of EPO levels
(i) Rudresh and Vivek [48] (ii) International Journal of Medical Research and Review (iii) 2016	Cross-sectional study	70 heart failure patients, age 54. 86 ± 11.75 years and 30 control, age 52.03 ± 13.21 years	—	The mean RDW in patient was 15.763 ± 2.609 and in controls was 13.17 ± 0.75, respectively	(i) Not ensure that RDW is independent of EPO levels
(i) Sotiropoulos et al. [49] (ii) ESC Heart Failure (iii) 2016	Prospective cohort	402 acute heart failure without acute coronary syndrome, age 64–86 years	1 year	(i) All-causemortality of all patients increased with quartiles of rising RDW (chi square 18; *p* < 0.001) (ii) In acute heart failure with LVEF ≥50%, the probability of all-cause mortality increased with rising RDW (*n* = 116; chi square 9.9; *p* = 0.0195)	(i) Without consideration of cardiovascular mortality or rehospitalization rate (ii) Echocardiograms were not obtained in all patients
(i) Liu et al. [50] (ii) Medical Science Monitor (iii) 2016	Retrospective analysis	179 chronic heart failure patients with different NYHA class, age 49–83 years	—	RDW increased significantly in class III and IV compared with class I (14.3 ± 2.3% and 14.3 ± 1.7% versus 12.9 ± 0.8%, *p* < 0.01)	(i) Only in-hospital baseline data were collected (ii) Healthy controls were not recruited

**Table 3:** Studies exploring association between red blood cell distribution width (RDW) and myocardial infarction (MI).

First author, journal, year	Study design	Study population	Mean follow-up	Major outcomes	Major limitations
(i) Tonelli et al. [20] (ii) Circulation (iii) 2008	Post hoc analysis	4111 participants with hyperlipidemia and a history of myocardial infarction, age 21–75 years	A median of 59.7 months	The top RDW quartile had a 56% increased risk of fatal coronary disease or nonfatal myocardial infarction when compared to subjects in the lowest quartile (HR 1.56, 95% CI 1.17–2.08)	(i) Not rule out the possibility of residual confounding (ii) The samples cannot not be representative of the general population
(i) Chen et al. [51] (ii) American Journal of Epidemiology (iii) 2010	Prospective cohort	3226 participants without history of stroke, coronary heart disease, or cancer, age>35 years	A median of 15.9 years	The highest RDW quartile was 1.46 for all-cause mortality compared with the lowest quartile (95% CI: 1.17–1.81)	(i) Few cases met the anemia criteria, which resulted in fairly wide confidence intervals (ii) Not reported data on specific causes of non-CVD death (iii) Only measured the RDW values once
(i) Zalawadiya et al. [52] (ii) American Journal of Cardiology (iii) 2010	Multiethnic cohort	7556 participants, age 41.5–15.8 years	10 years	The risk of being classified in the intermediate risk category of coronary heart disease was 53% greater (95% CI: 1.38–1.69, *p* < 0.001) with each unit increase in RDW value	(i) Actual cardiovascular events during a set follow-up period was unavailable
(i) Lee et al. [53] (ii) Clinical Cardiology (iii) 2013	Retrospective analysis	1596 patients with acute myocardial infarction, mean age, 64.5 ± 11.9 years	1634 ± 342 days	The RDW levels were significantly higher in patients with 12-month major adverse cardiac events (13.8 ± 1.3% versus 13.3 ± 1.2%, *p* < 0.001)	(i) Cannot exclude the possibility of residual confounding factors (ii) Not adjusted the RDW for nutrients (such as iron, folate, and vitamin B12)
(i) Arbel et al. [54] (ii) Thrombosis and Haemostasis (iii) 2014	Registry-based, retrospective cohort	225,006 subjects from health registry, age ≥ 40 years	5 years	Compared to patients with a RDW of 13% or lower, patients with RDW > 17% had a HR of 3.83 (95% CI: 3.12–4.69, *p* < 0.001) for all-cause mortality and 1.22 (95% CI: 1.04–1.42, *p* = 0.01) for major adverse cardiac events	(i) Not rule out the possibility of residual confounding (ii) Not reported data on specific causes of non-CVD death
(i) Skjelbakken et al. [125] (ii) Journal of the American Heart Association (iii) 2014	Prospective cohort	25,612 participants with no previous myocardial infarction, mean age 40.2–52.8 years	15.8 years	There was a linear association between RDW and risk of myocardial infarction, for which a 1% increment in RDW was associated with a 13% increased risk (HR 1.13; 95% CI: 1.07–1.19)	(i) The RDW measure was not repeated, there remained random measurement error (ii) Participants may underestimate the true prevalence of diabetes
(i) Sun et al. [22] (ii) Cardiology (iii) 2014	Prospective cohort	691 patients with STEMI, free of heart failure	41.8 months	High RDW was associated with all-cause mortality (HR: 3.43; 95% CI: 1.17–8.32; *p* = 0.025)	(i) Not rule out the possibility of residual confounding (ii) The sample size was relatively small
(i) Sahin et al. [126] (ii) Medical Principles and Practice (iii) 2015	Cross-sectional study	335 patients with NSTEMI, age 50–79 years	A median of 18 ± 11 months	The RDW levels of patients were significantly higher in the high SYNTAX group than in the low SYNTAX group (15.2 ± 1.8 versus 14.2 ± 1.2, *p* < 0.001)	(i) The sample size was relatively small (ii) Only measured hemoglobin levels, but not other factors such as iron, vitamin B12 and folate
(i) Sahin et al. [126] (ii) Clinics (iii) 2015	Cross-sectional study	251 adult patients with NSTEMI over a 1-year period, age >50 years	—	The RDW was higher in the group with non-ST-elevation myocardial infarction compared with the patient group with unstable angina (14.6 ± 1.0 versus 13.06 ± 1.7, resp., *p* = 0.006)	(i) The sample size was relatively small (ii) Only Hb levels were measured in the study

**Table 4:** Studies exploring association between red blood cell distribution width (RDW) and coronary atherosclerosis.

First author, journal, year	Study design	Study population	Mean follow-up	Major outcomes	Major limitations
(i) Yalçin et al. [127] (ii) European Journal of General Medicine (iii) 2012	Cross-sectional and observational study	296 stable eligible patients, 71% (mean age 61 ± 11 years) of them had coronary artery disease and 29% (mean age 52 ± 11 years) of them had normal coronary arteries	—	Red blood cell distribution width values were significantly different among the subgroups determined for the severity and extent of coronary artery disease	(i) The sample size was relatively small (ii) It does not explain the exact mechanism (iii) Control group included the patients who are not completely normal (iv) Not rule out the possibility of residual confounding
(i) Çelik et al. [128] (ii) American Journal of Cardiology (iii) 2014	Retrospective analysis	572 patients without coronary artery disease history	—	(i) RDW was found to be higher in patients with critical stenosis, than those without (13.63 ± 1.28 versus 14.31 ± 1.58, *p* < 0.001) (ii) RDW was an independent predictor of the severity of atherosclerotic lesions	(i) No follow-up of the patient (ii) The sample size was relatively small
(i) Chaikriangkrai et al. [129] (ii) Arteriosclerosis, Thrombosis, and Vascular Biology Arteriosclerosis, Thrombosis, and Vascular Biology (iii) 2014	Cross-sectional study	832 patients without known coronary artery disease who presented with acute chest pain, age > 18 years	10	No association between RDW and coronary calcification presence or severity	(i) Causal relationship cannot be established from this study design (ii) Patients not having CBC performed were excluded, which might create a bias

**Table 5:** Studies exploring association between red blood cell distribution width (RDW) and atrial fibrillation (AF).

First author, journal, year	Study design	Study population	Mean follow-up	Major outcomes	Major limitations
(i) Adamsson et al. [130] (ii) Journal of Internal Medicine (iii) 2013	Prospective cohort study	27,124 subjects from the general population with no history of atrial fibrillation, myocardial infarction, heart failure or stroke. Age 45–73 years	13.6 years	HR for incidence of atrial fibrillation was 1.33 (95% CI 1.16–1.53) for the fourth versus first quartile of RDW (*p* < 0.001)	(i) The study did not have information on HbA1c and lipid levels for the entire cohort (ii) Some cases of atrial fibrillation might be treated only in primary care and therefore would not be included in this study (iii) No detailed information about the AF cases at the time of diagnosis
(i) Chaikriangkrai et al. [131] (ii) African Health Sciences (iii) 2014	Retrospective analysis	(i) 63 hypertensive patients with atrial fibrillation, age 71.09 ± 8.50 years (ii) 63 hypertensive patients without atrial fibrillation, age 70.97 ± 8.24 years	—	RDW level was different among patients with atrial fibrillation and without atrial fibrillation (15.13 ± 1.58 and 14.05 ± 1.15, *p* < 0.01).	(i) The sample size was relatively small (ii) Echocardiographic parameters were not obtained concomitantly with blood sampling
(i) Güngör et al. [132] (ii) Journal of Thrombosis and Thrombolysis (iii) 2014	Retrospective study	117 non-valvular AF patients including 103 paroxysmal and 14 chronic atrial fibrillation, aged > 18 years	—	RDW (OR 4.18, 95% CI: 2.15–8.15; *p* = 0.01), hs-CRP (OR 3.76, 95% CI: 1.43–9.89; *p* = 0.01) and left atrial volume (OR 1.31, 95% CI: 1.06–1.21; *p* = 0.01) as the independent markers of non-valvular atrial fibrillation	(i) The study population was small (ii) The study did not assess specific markers of oxidative stress (iii) No detailed information about the AF cases at the time of diagnosis
(i) Gurses et al. [55] (ii) Journal of Interventional Cardiac Electrophysiology (iii) 2015	Prospective study	299 patients with symptomatic paroxysmal or persistent atrial fibrillation despite ≥1 antiarrhythmic drug(s), age 55.40 ± 10.60 years	24 months	(i) Patients with late atrial fibrillation recurrence had higher RDW levels (14.30 ± 0.93 versus 13.52 ± 0.93%, *p* < 0.001) (ii) RDW level was an independent predictor for late AF recurrence (HR 1.88, 95% CI: 1.41–2.50, *p* < 0.001)	(i) The study population was small (ii) Not illustrate the accurate mechanism
(i) Chaikriangkrai et al. [131] (ii) International Journal of Cardiology (iii) 2015	Pilot study	109 patients undergoing cardiac surgery, age 66.9 ± 9.5 years	—	RDW was the only independent predictor of postoperative atrial fibrillation (OR: 1.46; 95% CI: 1.078–1.994; *p* = 0.015). A RDW cut-off point of 13.35 was related to postoperative atrial fibrillation with a sensitivity of 80% and a specificity of 60%	(i) The study population was small (ii) The study did not assess specific markers of oxidative stress (iii) Only baseline values were recorded
(i) Korantzopoulos and Liu [133] (ii) Journal of Cardiology (iii) 2016	Retrospective study	101 patients with symptomatic SSS undergoing dual-chamberpacemaker implantation, median age 77 years	—	Left atrial diameter was increased in tachy-brady patients (44 mm versus 39 mm; *p* = 0.05). Also, the RDW was greater in these patients (14.7% versus 13.7%; *p* = 0.02)	(i) The study population was small (ii) The author did not assess the AF risk and atrial fibrillation burden after pacemaker implantation using the device diagnostics (iii) The author did not assess specific novel markers of oxidative stress and inflammation

**Table 6:** Studies exploring association between red blood cell distribution width (RDW) and primary hypertension.

First author, journal, year	Study design	Study population	Mean follow-up	Major outcomes	Major limitations
(i) Tanindi et al. [59] (ii) Blood Pressure (iii) 2012	Cross-sectional and observational study	128 patients with hypertension, 74 patients with prehypertension and 36 healthy controls, age >18 years	—	(i) Mean RDW values were 15.26 ± 0.82, 16.54 ± 0.91 and 13.87 ± 0.94 in prehypertensive, hypertensive and control groups, respectively (*p* < 0.05). (ii) Systolic and diastolic blood pressures were strongly correlated with RDW (*r* = 0.848 and *r* = 0.748, resp., *p* < 0.01)	(i) The sample size was relatively small (ii) Control group included the patients who are not completely normal (iii) Not rule out the possibility of residual confounding (iv) It does not explain the exact mechanism
(i) Sen-Yu et al. [134] (ii) Journal of Obstetrics and Gynaecology Research (iii) 2016	Retrospective study	149 pregnancies with pregnancy hypertension disease and 70 health pregnant women as controls, age>18 years	10 months	RDW in different gestational time (20th week, 24th week, 28th week) of different pregnant women groups had differences (*p* < 0.05), but pregnant women in the same group had no difference from 20th week to 28th week (*p* > 0.05)	(i) The sample size was relatively small (ii) Not rule out the possibility of residual confounding
(i) Reddy et al. [135] (ii) International Journal of Advances in Medicine (iii) 2016	Cross-sectional and observational study	200 patients and 100 controls, age >50 years	—	Hs-CRP levels and RDW levels are both equally effective as a predictive marker for hypertension	(i) No further statistical analysis (ii) The sample size was relatively small (iii) RDW value was measured only once
(i) Su et al. [136] (ii) BMJ Open (iii) 2016	Cross-sectional study	708 patients with essential hypertension, age 18–90 years	—	There was significantly increased RDW in reverse dippers (13.52 ± 1.05) than dippers (13.25 ± 0.85) of hypertension (*p* = 0.012)	(i) There was no a longer period of prospective observation providing more prognostic information (ii) The participants in our study are exclusively limited to northern Chinese patients from a single center (iii) They excluded 10 extreme dippers from the analysis in this study for their small sample size
(i) Özcan et al. [137] (ii) Blood Pressure (iii) 2013	Cross-sectional study	(i) 127 dipper patients, mean age, 52 ± 12 years (ii) 120 non-dipper patients, mean age, 54 ± 13 years	—	Nondippers had significantly higher RDW levels than dippers (14.6 versus 13.0, *p* < 0.001, resp.)	(i) It just represented a single-center experience (ii) Nondipper hypertensive patients were not followed-up in terms of future adverse cardiovascular events (iii) It does not explain the exact mechanism

**Table 7:** Studies exploring association between red blood cell distribution width (RDW) and carotid atherosclerosis and stroke.

First author, journal, year	Study design	Study population	Mean follow-up	Major outcomes	Major limitations
(i) Wen et al. [67] (ii) Experimental and Clinical Cardiology (iii) 2010	Cross-sectional study	156 hypertensive inpatients, age 60–85 years	—	A high baseline RDW was observed in patients with an increased IMT/ID ratio (95% CI: 4.54–28.59; *p* = 0.008) IMT means intimal-medialthickness ID means inner diameter	The sample size was relatively small
(i) Kaya et al. [138] (ii) Clinical and Applied Thrombosis/hemostasis (iii) 2013	Prospective study	153 patients with heart failure, age 56–76 years	1 year	An RDW ≥ 15.2% measured on admission had 87% sensitivity and 74% specificity in predicting stroke in patients with heart failure (area under the curve: 0.923, 95% CI: 0.852–0.994, *p* < 0.001)	(i) The sample size was relatively small (ii) Not make distinction between hemorrhagic and ischemic stroke (iii) The RDW may increase in many clinical situations, only the Hb level was checked in this study
(i) Wonnerth et al. [139] (ii) European Journal of Clinical Investigation (iii) 2014	Prospective, single-center, cross-sectional cohort	1286 patients with neurological asymptomatic carotid atherosclerosis	A median of 6.2 years	HR (1-SD increment of RDW) was 1.39 (95% CI: 1.27–1.53; *p* < 0.001) for all cause and 1.43 (95% CI: 1.28–160; *p* < 0.001) for cardiovascular mortality, respectively	(i) Data deriving from a post-hoc analysis of a prospective single-centerinvestigation, possible explanations for RDW variations cannot be addressed (ii) Not illustrate the accurate mechanism
(i) Vijayashree et al. [140] (ii) Archives of Medical Science Ams (iii) 2014	Retrospective cross-sectional study	236 patients hospitalized at the neurology ward, age 18–55 years	—	The mean RDW values of young patients with stroke were significantly higher than patients with epilepsy or multiple sclerosis (14.9 ± 1.2, 13.3 ± 1.2, 13.4 ± 0.6, *p* < 0.0001, resp.)	(i) The study was carried out in only one center (ii) Not illustrate the accurate mechanism
(i) Söderholm et al. [68] (ii) Plos One (iii) 2015	Population-based cohort study	26,879 participants without history of coronary events or stroke	A mean of 15.2 years	(i) Incidences of total cerebral infarction (*n* = 1,544) and stroke (*n* = 1,869) were both increased in individuals with high RDW (ii) RDW was positively associated with intima-media thickness of the common carotid artery (*p* = 0.011)	(i) The numbers of intracerebral hemorrhage and subarachnoid hemorrhage cases were considerably lower (ii) Not illustrate the accurate mechanism
(i) Güçlü et al. [141] (ii) Turkish Nephrology Dialysis and Transplantation Journal (iii) 2016	Post hoc analysis	(i) 30 healthy controls, age 52.76 ± 13.57 years (ii) 30 patients with chronic kidney disease, age 52.28 ± 13.75 years (iii) 37 hemodialysis patients, age 56.02 ± 16.06	—	The RDW value was higher in the predialysis group than controls with a trend to statistical significance (*p* = 0.067). RDW value showed positive correlation with intima-media thickness (*r* : 0.356*p* = 0.012) and CRP (*r* : 0.361*p* = 0.004)	(i) The sample size was relatively small (ii) Not illustrate the accurate mechanism
(i) Lappegård et al. [142] (ii) Thrombosis and Haemostasis (iii) 2016	A single-center prospective, population-based study	25,992 participants, age > 25 years	A mean of 15.8 years	HR for higher risk of stroke (1-SD increment of RDW): 1.13 (95% CI, 1.07–1.20)	(i) There was only one measurement of RDW throughout the study period (ii) Residual confounding cannot be completely ruled out
(i) Ren et al. [143] (ii) BMC Cardiovascular Disorders (iii) 2017	Cross-sectional study	803 patients with metabolic syndrome undergoing carotid ultrasonography examination, age 24 to 54 years	—	Compared with the first quartile, people with third and fourth quartile level gave obvious higher risk of carotid artery atherosclerotic trend (OR = 1.41, 95% CI: 1.01–197; OR = 2.10, 95% CI: 1.30–3.40)	(i) The cross-sectional design limits the causal relationship between RDW and carotid intimal-medial thickness (ii) Only metabolic syndrome patients were included (iii) Not include drug history in the final analysis (iv) Not illustrate the accurate mechanism

## Additional Points

*Core tips*. This review explores the available data in the literatures concerning the association between red blood cell distribution width and cardiovascular and cerebrovascular diseases, as well as the underlying mechanisms.
